# Bible Argument against Criminal Abortions

**Published:** 1859-04

**Authors:** 


### *Bible Argument against Criminal Abortions.*

DEAR SIR:—The writer is grateful for your faithful and timely discussion of the subject of "Criminal Abortions;" and he does not think that you will esteem this testimony of less importance, when he informs you, that for more than a quarter of a century, he has tried to preach the gospel.

You will allow me to mention to you a bible argument which may aid you and your associates in the needful reform in which you are enlisted. I refer to

MALACHI II: 15.—"And did he not make one? Yet had he the residue of the Spirit. And wherefore *one? That he might seek a Godly seed.*"

The inspired prophet is reproving each transgressor of the marriage vow for ill treating "the wife of thy covenant." He refers these sinners to the original institution of marriage. "And did he not make one?" That is—one wife for Adam. "Yet had he the residue of the Spirit;" he might have created a plurality of wives, if he had thought best.

"And wherefore *one? That he might seek a Godly seed.*"

Here the great object of marriage is made prominent.

The family relation is the foundation of all society. To "seek a Godly seed," is the reason why God has arranged that the human race shall exist in successive generations, and in well guarded family enclosures.

Hence, we infer—that those who encourage criminal abortions—do defy Jehovah in respect to his great design in the establishment of the "Holy Covenant of Marriage." Their conduct tends to subvert an institution which is at the foundation of the perpetuity of the human race, and is essential to the securing of the religious education of children as the means of perpetuating genuine religion.

Yours, truly,

ANDOVER, N. Y., Feb. 12, '59. J. K. JOHNSON.

We received the above communication in time for our last number, but it was inadvertently omitted. We were glad to receive it as indicating the interest which is felt by our correspondent, who is not of our profession, in the subject of criminal abortions, and were gratified to see that our Journal is sometimes read by laymen. Though we write for the profession and devote our labors entirely to its advancement, there are things which appear in Medical Journals which should come under the eye of the public; and especially we would desire the public to be alive to the enormity of criminal abortion, which as every physician knows, not one in a hundred appreciate in the slightest degree; not even those who might be said to have a high sense of morality. We venture to hope, however, that at the coming sitting of the National Medical Association, some means will be devised for bringing this matter before the public and attempting its reform. Certainly, in no regard, does a christian society more need the assistance of its medical men.

